# Error Analysis of Heterodyne Interferometry Based on One Single-Mode Polarization-Maintaining Fiber

**DOI:** 10.3390/s23084108

**Published:** 2023-04-19

**Authors:** Yibin Qian, Jiakun Li, Qibo Feng, Qixin He, Fei Long

**Affiliations:** Key Laboratory of Luminescence and Optical Information, Ministry of Education, Beijing Jiaotong University, Beijing 100044, China

**Keywords:** laser measurement, polarization-maintaining fiber, simulation

## Abstract

Using polarization-maintaining fiber (PMF) in dual-frequency heterodyne interferometry has the advantages of reducing the laser’s own drift, obtaining high-quality light spots, and improving thermal stability. Using only one single-mode PMF to achieve the transmission of dual-frequency orthogonal, linearly polarized beam requires angular alignment only once to realize the transmission of dual-frequency orthogonal, linearly polarized light, avoiding coupling inconsistency errors, so that it has the advantages of high efficiency and low cost. However, there are still many nonlinear influencing factors in this method, such as the ellipticity and non-orthogonality of the dual-frequency laser, the angular misalignment error of the PMF, and the influence of temperature on the output beam of the PMF. This paper uses the Jones matrix to innovatively construct an error analysis model for the heterodyne interferometry using one single-mode PMF, to realize the quantitative analysis of various nonlinear error influencing factors, and clarify that the main error source is the angular misalignment error of the PMF. For the first time, the simulation provides a goal for the optimization of the alignment scheme of the PMF and the improvement of the accuracy to the sub-nanometer level. In actual measurement, the angular misalignment error of the PMF needs to be smaller than 2.87° to achieve sub-nanometer interference accuracy, and smaller than 0.25° to make the influence smaller than ten picometers. It provides theoretical guidance and an effective means for improving the design of heterodyne interferometry instruments based on PMF and further reducing measurement errors.

## 1. Introduction

A dual-frequency heterodyne laser interferometer plays an irreplaceable role in the field of precision measurement. With its excellent anti-interference performance and measurement accuracy, it is widely used in the measurement and error correction of high-end equipment, such as high-precision machine tools and lithography machines [1,2,3,4]. For a traditional dual-frequency heterodyne laser interferometer, the error caused by the heating of the laser is difficult to ignore, so the laser and the measuring device are usually placed far apart to isolate the heat source. In order to minimize the impact of the environmental interference on the way from the laser to the measuring device, a PMF is usually used to connect the laser and the measurement device. In 2011, J. D. Ellis et al. used two PMFs to couple Delft interferometers, and the two fibers, respectively, transmitted polarized beams of a single frequency to construct a fiber–optic interferometry system, realizing sub-nanometer measurements [5]. In 2014, S. R. Gillmer et al. used two PMFs to couple the acousto-optic modulation dual-frequency laser to realize the three-degree-of-freedom measurement of displacement, pitch angle, and yaw angle [6]. In 2004, B.A.W.H. Knarren et al. used one PMF coupled with a dual-frequency laser to prove the feasibility of one PMF coupled with a dual-frequency laser for heterodyne measurement, and tested the error effects of different performance fibers on interferometric measurements [7]. In 2016, Q. Feng et al. proposed a new method for the simultaneous measurement of six-degree-of-freedom geometric motion errors. For the first time, a polarization-maintaining fiber-coupled dual-frequency laser was used to simultaneously measure six-degree-of-freedom geometric motion errors of linear guides [8]. The dual-frequency heterodyne laser interferometer uses the phase change in the photodetector to measure the displacement. However, the polarization state and orthogonality of the laser beam source are not ideal, the coupling of the PMF is not good, and the extinction ratio is low. These questions cause the phenomenon of polarization mixing, which in turn produces a large nonlinear error. At the same time, in the case of poor PMF coupling, environmental factors, such as temperature and stress, will have a greater impact on the polarization state of the laser, making it difficult to accurately identify phase changes and greatly reducing the measurement accuracy. In order to improve the measurement accuracy of the heterodyne interferometer based on PMF, it is necessary to model and analyze various factors that may cause nonlinear errors and clarify the influence of each factor on nonlinear errors. In recent years, domestic and foreign scholars have never stopped studying the nonlinear error of heterodyne interferometry. In 1996, Chien-ming Wu et al. found that the nonlinear error of heterodyne interferometry comes from the mutual crosstalk of beams at different frequencies, in which the first-order nonlinear error can be compensated, and the second-order nonlinear error is difficult to eliminate [9]. In 2006, Wenmei Hou et al. found that the measurement accuracy of heterodyne interferometry is also affected by the nonlinear drift of the inherent system, and proposed a detection method to compensate it after separating it from the thermal drift [10]. In 2010, Hongfang Chen et al. achieved a reduction of the first harmonic of the nonlinear error to 40% of the original by measuring the axial rotation angle of the corner cube [11]. In 2015, Liping Yan et al. studied the nonlinear error caused by the misalignment of the polarization beam splitter (PBS) in the heterodyne interferometer, and the results showed that the nonlinear error caused by the yaw angle error of the PBS was the largest [12]. Optical methods focus on compensating for a single factor that introduces nonlinear errors. In addition to using optical methods, such as adjusting the optical path structure or adding optical devices to study and compensate nonlinear errors, compensation through numerical methods, such as algorithms or signal processing, can compensate multiple errors at one time. For example, in 2021, Yanqi Zhang et al. proposed that a noise estimation subtraction algorithm can improve the sensitivity of the heterodyne laser interferometer by an order of magnitude, reducing the noise of nonlinear optical pathlength noise, laser frequency noise, and temperature fluctuations in heterodyne laser interferometers [13]. Moreover, a variety of compensation algorithms, such as ellipse-matching and neural network methods, have been proposed [14,15,16,17,18,19]. These studies provide an important reference for the error analysis of heterodyne interferometry based on PMF. From the above research, it can be seen that in the aspect of using PMF-coupled dual-frequency lasers for heterodyne measurement, past research mainly focused on the measurement of two PMFs coupled with single-frequency light [5,6]. In the study of heterodyne measurement based on one single PMF, the errors caused by the laser’s own defects and fiber misalignment have not been fully analyzed [7,8]. Moreover, the analysis of nonlinear errors in heterodyne interferometry rarely involves heterodyne measurement systems based on one single PMF [9,10,11,12]. Therefore, further research is needed. In this respect, this paper innovatively constructs a heterodyne interferometry error model based on one single-mode PMF, and uses the Jones matrix to analyze the various factors that cause nonlinear errors in the heterodyne interferometry based on one single-mode PMF. We comprehensively analyzed how the ellipticity $\rho_{1},\;\;\rho_{2}$ and non-orthogonality β of the dual-frequency laser, the angular misalignment error θ of the PMF, and the temperature T influence the nonlinear error. It is clarified that the angular misalignment error is the main factor affecting the nonlinear error in the heterodyne interferometry based on the PMF, which provides theoretical guidance for improving the design of the heterodyne interferometry instrument based on one single-mode PMF, further reducing the measurement error. For the first time, the simulation provides a goal for the optimization of the alignment scheme of the PMF and the improvement of the accuracy to the sub-nanometer level or even better. In actual measurement, the angular misalignment error of the PMF needs to be smaller than 2.87° to achieve sub-nanometer interference accuracy, and smaller than 0.25° to make the influence smaller than ten picometers. It provides an efficient method for analyzing nonlinear errors in the instrument’s actual measurement. In terms of error compensation, this paper not only provides guidance for optical methods through simulation, and points out the importance of alignment of PMF and the basis for alignment requirements, but also provides formulas for numerical methods to compensate for errors and calculate non-linear errors produced by multiple factors. Alongside that, it provides a new method for improving the accuracy of heterodyne interferometry in the fields of high-precision numerical control machine tools, chips, and circuit manufacturing and processing.

## 2. Error Model of Heterodyne Interferometry Based on One Single-Mode PMF

For an ideal dual-frequency orthogonal laser, its outgoing laser beam consists of two orthogonal, linearly polarized components with slightly different frequencies (the frequency difference range is generally from 1 MHz to 40 MHz); however, in practical applications, the two components of the outgoing laser beam exhibit small ellipticity and non-orthogonality, which will lead to inevitable mode mixing when passing through the PMF. This will also bring nonlinear errors to subsequent measurements [20,21].

**Figure 1:**
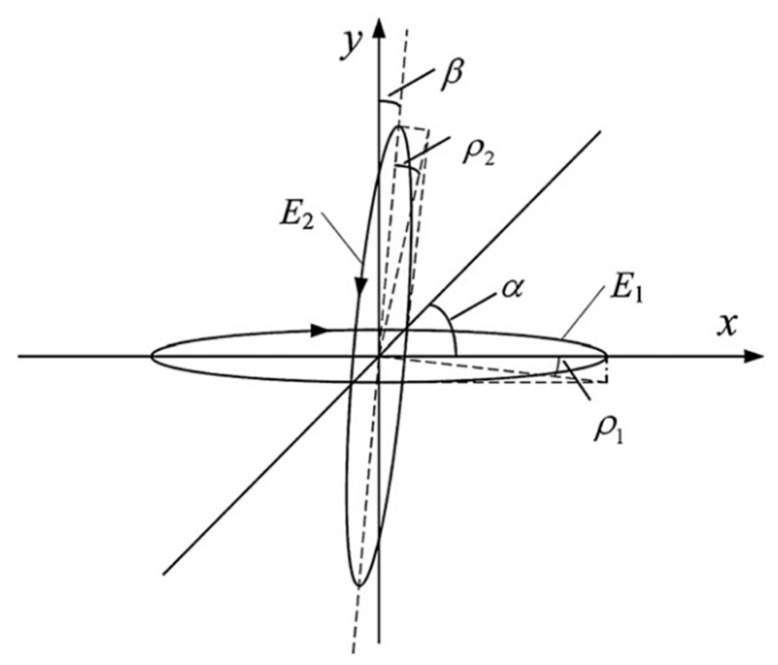
Schematic diagram of the polarization state of the output beam of a dual-frequency laser.

As shown in Figure 1 [20], the output beam of the laser is composed of two non-orthogonal elliptically polarized components, ${\hat{E}}_{1}$ and ${\hat{E}}_{2}$, the frequencies are $f_{1}$ and $f_{2}$, respectively, the ellipticities are $\rho_{1}$ and $\rho_{2}$, respectively, and the degree of non-orthogonality between them is β.

Assuming that the x-axis of the coordinate system is parallel to ${\hat{E}}_{1}$, the Jones matrix of ${\hat{E}}_{1}$ is as follows:

(1) $${\hat{E}}_{1}=a_{1}\cdot \left[\begin{array}{c} cos\rho_{1}\exp\left[i\left(2\pi f_{1}t\right)\right]\\ sin\rho_{1}\exp\left[i\left(2\pi f_{1}t+\pi /2\right)\right] \end{array}\right]$$

For convenience in the analysis, the normalized Jones matrix of $E_{1}$ is:

(2) $${\hat{E}}_{1}=\left[\begin{array}{c} cos\rho_{1}\\ isin\rho_{1} \end{array}\right]$$

The normalized Jones matrix of ${\hat{E}}_{2}$ is:

(3) $${\hat{E}}_{2}=\left[\begin{array}{cc} sin\beta & -cos\beta \\ cos\beta & sin\beta \end{array}\right]\left[\begin{array}{c} \cos\left(90{}^{\circ}-\rho_{2}\right)\\ isin\left(90{}^{\circ}-\rho_{2}\right) \end{array}\right]=\left[\begin{array}{cc} sin\beta & -cos\beta \\ cos\beta & sin\beta \end{array}\right]\left[\begin{array}{c} sin\rho_{2}\\ -icos\rho_{2} \end{array}\right]\;=\left[\begin{array}{c} sin\beta sin\rho_{2}+icos\beta cos\rho_{2}\\ cos\beta sin\rho_{2}-isin\beta cos\rho_{2} \end{array}\right]$$

The Jones matrix of PMF is $D=\left[\begin{array}{cc} e^{i\Delta L} & 0\\ 0 & 1 \end{array}\right]$, where Δ is the birefringence and *L* is the fiber length. When the polarized ${\hat{E}}_{1}$ of the dual-frequency laser is aligned with the slow axis of the PMF, the outgoing beam of the PMF is:

(4) $${\hat{E}}_{1^{\prime }}=\left[\begin{array}{cc} e^{i\Delta L} & 0\\ 0 & 1 \end{array}\right]\left[\begin{array}{c} cos\rho_{1}\\ isin\rho_{1} \end{array}\right]=\left[\begin{array}{c} cos\rho_{1}e^{i\Delta L}\\ isin\rho_{1} \end{array}\right]\;{\hat{E}}_{2^{\prime }}=\left[\begin{array}{cc} e^{i\Delta L} & 0\\ 0 & 1 \end{array}\right]\left[\begin{array}{c} sin\beta sin\rho_{2}+icos\beta cos\rho_{2}\\ cos\beta sin\rho_{2}-isin\beta cos\rho_{2} \end{array}\right]=\left[\begin{array}{c} \left(sin\beta sin\rho_{2}+icos\beta cos\rho_{2}\right)e^{i\Delta L}\\ cos\beta sin\rho_{2}-isin\beta cos\rho_{2} \end{array}\right]$$

Usually, a half-wave plate is used to align the dual-frequency laser and the PMF. During the alignment process, there is inevitably an alignment error between the orthogonal linearly polarized light emitted by the dual-frequency laser and the fast and slow axes of the PMF, that is, the angular misalignment error *θ* of the PMF. Considering this error, the outgoing beam of the PMF is:

(5) $${\hat{E}}_{1^{\prime }}=\left[\begin{array}{cc} e^{i\Delta L} & 0\\ 0 & 1 \end{array}\right]\left[\begin{array}{cc} cos2\theta & sin2\theta \\ sin2\theta & -cos2\theta \end{array}\right]\left[\begin{array}{c} cos\rho_{1}\\ isin\rho_{1} \end{array}\right]\;{\hat{E}}_{2^{\prime }}=\left[\begin{array}{cc} e^{i\Delta L} & 0\\ 0 & 1 \end{array}\right]\left[\begin{array}{cc} cos2\theta & sin2\theta \\ sin2\theta & -cos2\theta \end{array}\right]\left[\begin{array}{c} sin\beta sin\rho_{2}+icos\beta cos\rho_{2}\\ cos\beta sin\rho_{2}-isin\beta cos\rho_{2} \end{array}\right]$$

**Figure 2:**
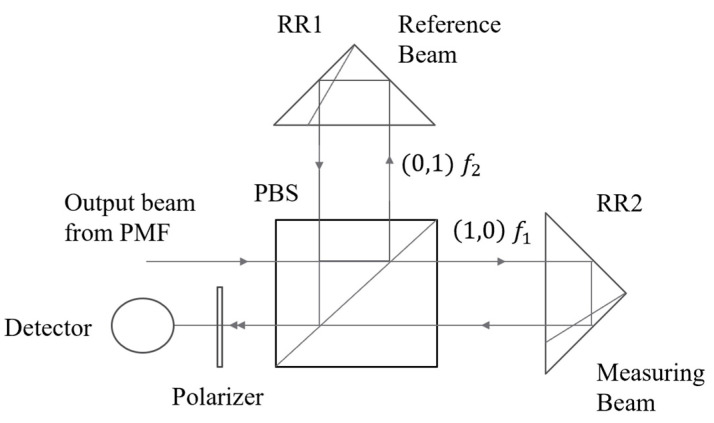
Schematic diagram of the reference beam and the measurement beam.

Taking ${\hat{E}}_{1^{\prime }}$ as measuring beam and ${\hat{E}}_{2^{\prime }}$ as reference beam, as shown in Figure 2, the above formula can be written as follows:

(6) $${\hat{E}}_{1^{\prime }}=\left[\begin{array}{c} A\\ a \end{array}\right]\;{\hat{E}}_{2^{\prime }}=\left[\begin{array}{c} B\\ b \end{array}\right]$$

In the formula, A and B are the amplitudes of the measuring beam and the reference beam, and a and b are the amplitudes of the crosstalk of the measuring beam and the crosstalk of the reference beam; that is, the amplitude of the beam coupled from its alignment axis to another axis.

After finishing, ${\hat{E}}_{1^{\prime }}$ and ${\hat{E}}_{2^{\prime }}$ can be written as:

(7) $${\hat{E}}_{1^{\prime }}=\left[\begin{array}{c} A\\ a \end{array}\right]=\left[\begin{array}{c} e^{i\Delta L}\left(cos2\theta cos\rho_{1}+isin2\theta sin\rho_{1}\right)\\ sin2\theta cos\rho_{1}-icos2\theta sin\rho_{1} \end{array}\right]\;{\hat{E}}_{2^{\prime }}=\left[\begin{array}{c} B\\ b \end{array}\right]=\left[\begin{array}{c} e^{i\Delta L}\left[\sin\left(2\theta +\beta \right)sin\rho_{2}+i\cos\left(2\theta +\beta \right)cos\rho_{2}\right]\\ -\cos\left(2\theta +\beta \right)sin\rho_{2}+i\sin\left(2\theta +\beta \right)cos\rho_{2} \end{array}\right]$$

In the actual measurement, the main source of nonlinear error is the frequency mixing of the outgoing beam. The measurement beam and reference beam can be expressed as [22,23]:

(8) $$\{\begin{array}{l} E_{1}=Acos\left(\omega_{1}t+\Delta \varphi \right)+bcos\left(\omega_{2}t+\Delta \varphi \right)\\ E_{2}=Bcos\left(\omega_{2}t\right)+acos\left(\omega_{1}t\right) \end{array}$$

In the formula, $\Delta \varphi =2\pi f_{d}$ is the measurement phase, $\omega_{1}$ and $\omega_{2}$ are the angular frequencies of the measurement beam and the reference beam. $f_{d}$ is the Doppler frequency shift introduced by the movement of the measuring target mirror. The measurement signal formed after the interference of the two is:

(9) $$\begin{array}{c} I_{m}\propto ABcos\left(\Delta \omega t+\Delta \varphi \right)+\left(Ab+Ba\right)cos\left(\Delta \omega t\right)+\\ abcos\left(\Delta \omega t-\Delta \varphi \right). \end{array}$$

In the formula, $\Delta \omega =\omega_{1}-\omega_{2}$. After transforming the above formula, we can get:

(10) $$I_{m}\propto ABcos\left(\Delta \omega t+\Delta \varphi +\Delta \varphi_{non}\right)$$

The nonlinear error $\Delta \varphi_{non}$ can be expressed as:

(11) $$\Delta \varphi_{non}=\tan^{-1}\left[\frac{-\left(Ab+Ba\right)\sin\varphi -ab\sin2\varphi }{AB+\left(Ab+Ba\right)\cos\varphi +ab\cos2\varphi }\right]$$

Generally speaking, the ratio of the crosstalk of the reference beam and the measurement beam in the heterodyne measurement is much smaller than 1 ($\frac{a}{A}\ll 1,\frac{b}{B}\ll 1$). Therefore, the above formula can be approximated as:

(12) $$\Delta \varphi_{non}\approx -\frac{Ab+Ba}{AB}\sin\Delta \varphi -\frac{ab}{AB}\sin2\Delta \varphi$$

In heterodyne measurement, the phase difference between the measurement beam and the reference beam is:

(13) $$\Delta \varphi =\frac{4\pi L}{\lambda }$$

Therefore, the nonlinear error of displacement measurement can be expressed as:

(14) $$\Delta L_{non}=\frac{\lambda }{4\pi }\Delta \varphi_{non}=-\frac{\lambda }{4\pi }(\frac{Ab+Ba}{AB}\sin\Delta \varphi +\frac{ab}{AB}\sin2\Delta \varphi )$$

A, a, B, and b in Equation (7) can be substituted into Equation (14) to get the nonlinear error of the displacement measurement.

In this section, the relationship between the nonlinear error of the system and the ellipticity $\rho_{1}$, $\rho_{2}$ and non-orthogonality β of the dual-frequency laser, the angular misalignment error θ of the PMF, and the birefringence Δ of the PMF are deduced, where the birefringence Δ of the PMF is affected by the change in temperature, T, and the numerical relationship between the two can be obtained when the parameters of the PMF are determined. Through the mathematical model derived in this section, the error of heterodyne interferometry based on one single-mode PMF can be analyzed to clarify the main influencing factors, and provide a theoretical basis for optimizing the design of the measurement system and improving the accuracy of heterodyne interferometry.

## 3. Analysis of the Nonlinear Error Simulation

**Figure 3:**
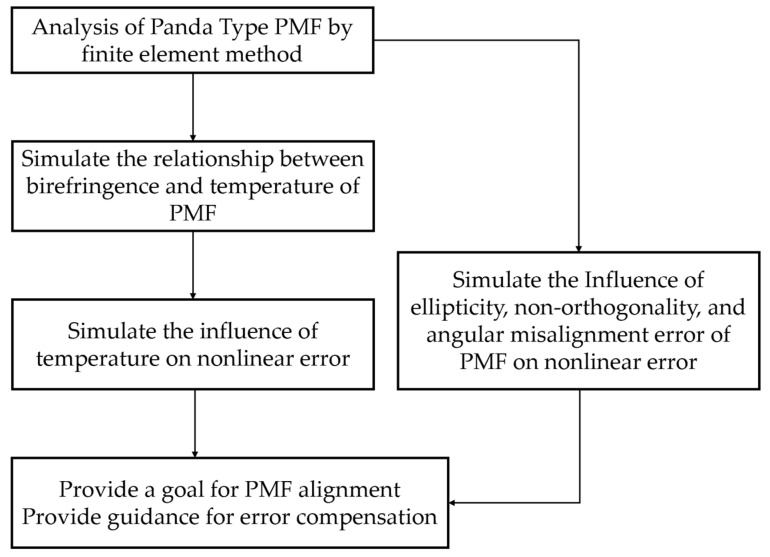
Nonlinear error simulation analysis flowchart.

According to the heterodyne interferometry error model based on one single-mode PMF derived in the previous section, the influence of each variable on the nonlinear error of the system can be simulated under the condition of controlling variables. The nonlinear error simulation analysis process is shown in Figure 3.

Before studying the relationship between temperature, T, and nonlinear error through simulation, it is first necessary to obtain the corresponding relationship between the birefringence Δ of the PMF and the temperature, T.

**Figure 4:**
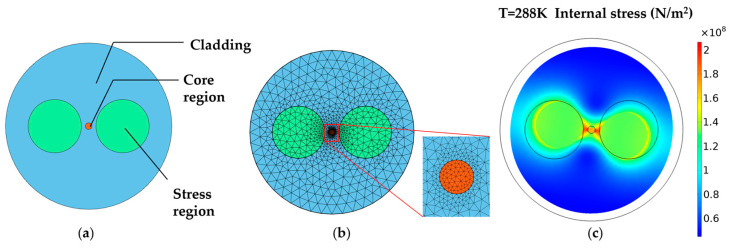
(**a**) Section of the Panda-type PMF. (**b**) Analysis of the Panda-type PMF by finite element method. (**c**) Internal stress of PMF at 288 K.

In this paper, the finite element method is used to construct the PMF, as shown in Figure 4, and the corresponding relationship between its birefringence Δ and temperature, T, is obtained [24,25,26]. The PMF is a common Panda-type PMF. The cladding material is pure SiO_2_, the core material is pure SiO_2_ doped with a small amount of GeO_2_, and the stress region is doped with B_2_O_3_. The fiber parameters are shown in Table 1.

**Figure 5:**
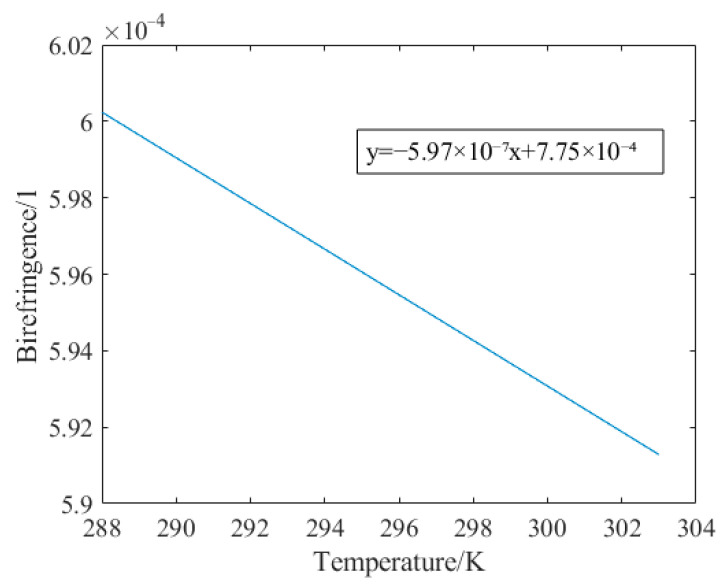
Simulation diagram of the relationship between birefringence and temperature of the PMF.

In order to simulate the relationship between temperature and birefringence in PMF, the annealing temperature of the optical fiber is set to 1020 °C. The temperature change in the PMF model is carried out from 288 K to 303 K, and the simulated corresponding relationship between the birefringence Δ and the temperature, T, of the PMF can be obtained, as shown in Figure 5.

Through the simulation of the PMF model, it can be seen that the birefringence of the polarization-maintaining fiber has a linear relationship with the temperature, in theory. After obtaining the relationship between the birefringence Δ of the PMF and the temperature, T, the influence of temperature on the nonlinear error can be calculated. Next, this paper will simulate the influence of the temperature, T, the ellipticity $\rho_{1}$, $\rho_{2}$ and non-orthogonality β of the dual-frequency laser, and the angular misalignment error θ of the PMF on the nonlinear error.

### 3.1. Influence of Temperature on the Nonlinear Error

**Figure 6:**
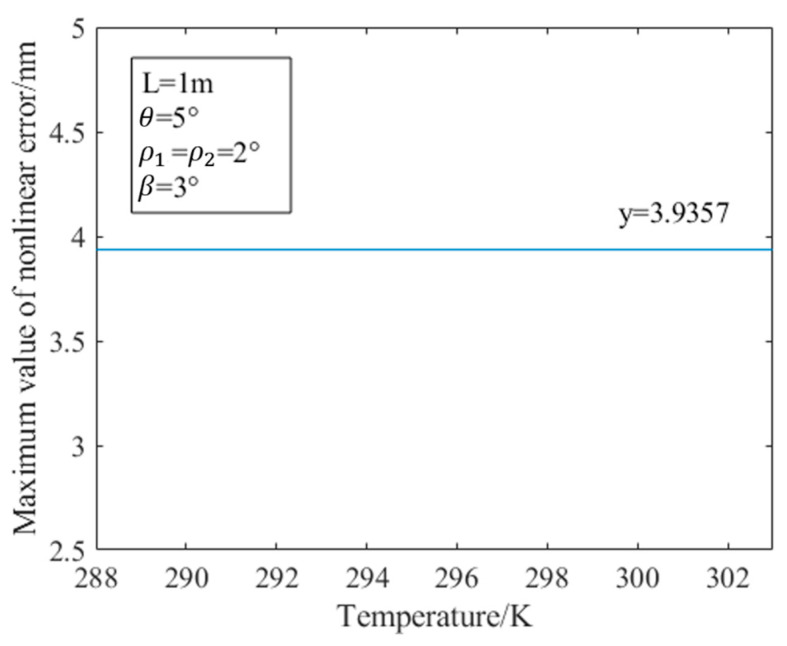
Simulation result of the influence of temperature change on nonlinear error.

**Figure 7:**
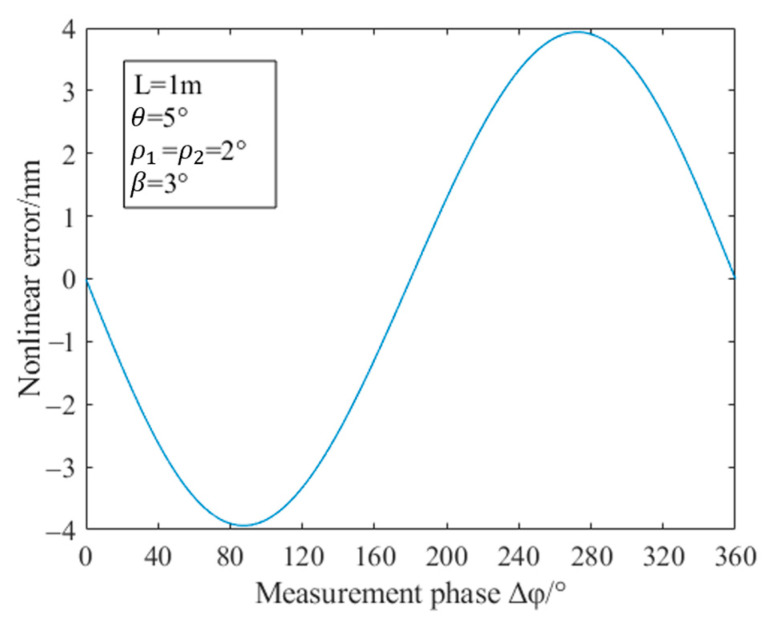
Relationship between the nonlinear error and the measurement phase under theoretical conditions.

In practical applications, the outgoing beam of the dual-frequency laser cannot be completely orthogonal and linear. There will always be an angular misalignment error in the PMF as well. Therefore, temperature changes affect the birefringence of the PMF, which in turn affects the nonlinearity error. The following analyzes the nonlinear error introduced by the birefringence change in the PMF caused by the temperature change: set the length of the PMF to 1 m; the ellipticity $\rho_{1}$ and $\rho_{2}$ of the dual-frequency laser are both 2°; the degree of non-orthogonality β is 3°; the angular misalignment error θ of the PMF is 5°; and the control temperature rises from 288 K to 303 K. The relationship between the temperature and the maximum value of the nonlinear error obtained by the simulation is shown in Figure 6, where the maximum value of the nonlinear error refers to the maximum value of the nonlinear error of the displacement measurement when the measurement phase changes. As shown in Figure 7, the nonlinear error of displacement measurement varies with the measurement phase. Since the results of the heterodyne measurement are calculated through the measurement phase, we mainly study the maximum value in nonlinear error analysis. In Figure 6, the maximum value of the nonlinear error obtained by simulation does not change with temperature, which is always 3.9357 nm. Next, this paper will explain the reasons for the simulation results.

The beat length $L_{p}$ of the PMF can be expressed as:

(15) $$L_{p}=\frac{\lambda }{\Delta }$$

The measurement phase, Δφ, can be expressed as:

(16) $$\Delta \varphi =\Delta \varphi_{1}+\Delta \varphi_{2}$$

where $\Delta \varphi_{1}$ is the phase difference inside the PMF, and $\Delta \varphi_{2}$ is the phase difference outside the PMF. When the laser beam passes through a beat length in the PMF, the magnitude of the nonlinear error changes in a cycle. The phase $\Delta \varphi_{1}$ in the PMF can be expressed as:

(17) $$\Delta \varphi_{1}=\left(n+\frac{L-nL_{p}}{L_{p}}\right)\times 360{}^{\circ}$$

**Figure 8:**
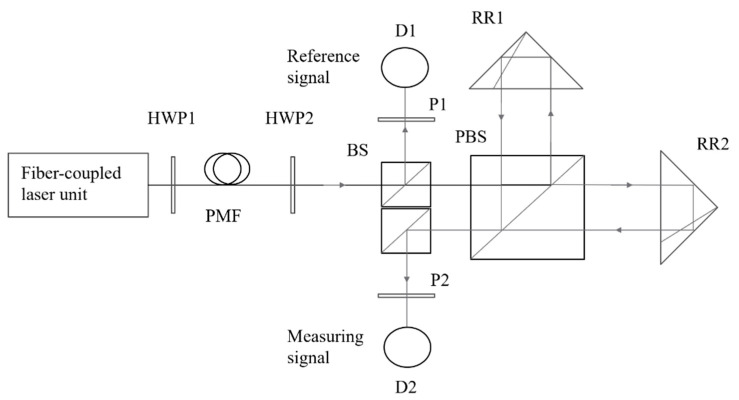
Schematic of a heterodyne measurement system based on one single-mode PMF. A reference signal detector is placed after the fiber to get rid of the phase error caused by the fiber.

The temperature change causes the beat length $L_{p}$ to change, which eventually leads to a change in $\Delta \varphi_{1}$. However, this change does not affect the maximum value of the sinusoidal curve, so it has no effect on the maximum value of the nonlinear error. This means that we do not need to consider the influence of the temperature change in the PMF on the nonlinear error in the working temperature range in the experiment. On the other hand, the heterodyne measurement system based on one single-mode PMF can subtract the phase error introduced by the temperature change in the PMF by placing the reference signal after the PMF, as shown in Figure 8.

After the orthogonal, linearly polarized beams pass through the PMF, the outgoing beams are split by the BS, and the reflected beams interfere with each other on the photodiode D1 after passing through the polarizer P1. The angle between the polarizer P1 and the orthogonal, linearly polarized beams should be adjusted at 45°. At this time, the interference intensity is the largest, and the reference signal can be expressed as:

(18) $$I_{1}=\frac{A_{0}^{2}}{2}cos\left[2\pi \left(f_{1}-f_{2}\right)t+\Delta \varphi_{1}\right]$$

After the transmitted beam of the BS is split by the PBS, the two orthogonal, linearly polarized beams are used as the reference beam and the measurement beam of the heterodyne interference, and then they are reflected by the reference mirror, RR1, and the measurement mirror, RR2, respectively, and return to the PBS. At this time, the phase difference between the two is $\Delta \varphi_{2}$. Interference on the photodiode D2 should be carried out after adjusting the polarizer P2 and the orthogonal, linearly polarized beams to 45°. At this time, the interference intensity is the largest, and the measurement signal can be expressed as:

(19) $$I_{2}=\frac{A_{0}^{2}}{2}cos\left[2\pi \left(f_{1}-f_{2}\right)t+\Delta \varphi_{1}+\Delta \varphi_{2}\right]$$

The above formula shows that the reference signal, $I_{1}$, and the measurement signal, $I_{2}$, are sinusoidal signals with equal periods and a phase difference of $\Delta \varphi_{2}$. According to the principle of the heterodyne measurement:

(20) $$\Delta \varphi_{2}=\frac{4\pi L}{\lambda }$$

Since the reference signal is placed behind the PMF, it can be seen that the change in $\Delta \varphi_{1}$ due to the temperature change in the PMF will not affect the measurement error.

### 3.2. Influence of Ellipticity Non-Orthogonality and Angular Misalignment Error of the PMF on the Nonlinear Error

**Figure 9:**
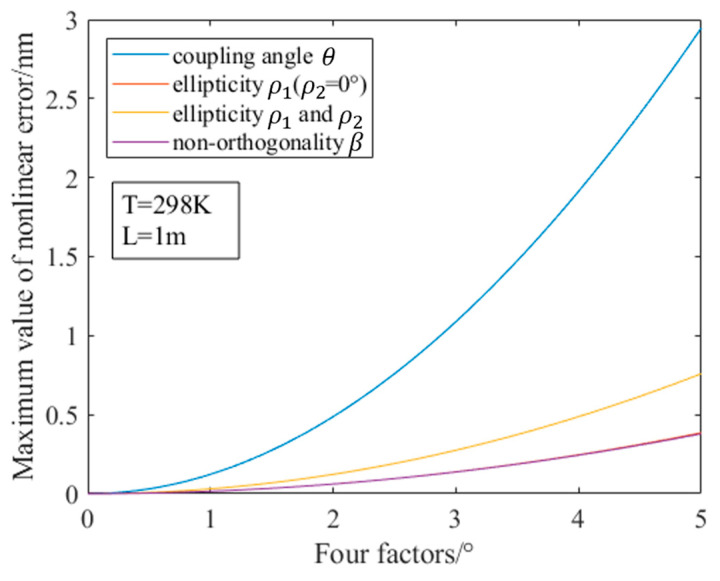
Comparison chart of the influence of the four influence factors on nonlinear error.

In the simulation, the temperature should first be set to 298 K. The length of the PMF is 1 m, and the wavelength of the dual-frequency laser is 632.8 nm. In this case, the control variable method is used to control the four influencing factors of the ellipticity $\rho_{1}$, $\rho_{2}$ and non-orthogonality β of the dual-frequency laser, and the angular misalignment error θ of the PMF. In the case of controlling other variables to 0°, we change a single variable from 0° to 5° (which covers most of the margin of error) to obtain the influence of the four influencing factors on the size of the nonlinear error. The simulation results are shown in Figure 9.

**Figure 10:**
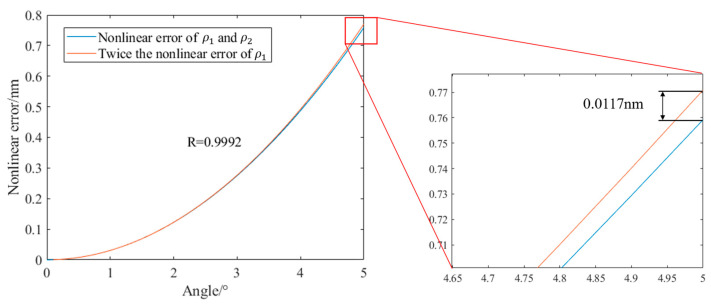
Comparison of the nonlinear errors between one ellipticity and two ellipticities of dual-frequency lasers.

It can be seen from Figure 9 that in the range from 0° to 5°, the angular misalignment error θ of the PMF has the greatest influence on the nonlinear error in the measurement. When the angular misalignment error reaches 5°, the nonlinear error is as high as 2.9469 nm. The non-orthogonality β of the dual-frequency laser has a similar effect on the nonlinear error as that caused by the ellipticity of one of the dual-frequency orthogonal beams. When one of the dual-frequency orthogonal beams is linearly polarized and the other has an ellipticity of 5°, the nonlinear error is 0.3854 nm. When the two orthogonal beams are both linearly polarized and there is a non-orthogonality of 5° between them, the nonlinear error is 0.3796 nm. It can be seen from Table 2 that both are much smaller than the nonlinear error caused by the angular misalignment error of the PMF. When the ellipticities $\rho_{1}$ and $\rho_{2}$ of the two beams of the dual-frequency laser exist at the same time, compared with only one beam having ellipticity and the other beam being linearly polarized, as shown in Figure 10, the nonlinear error is nearly doubled. Because the correlation coefficient R is 0.9992, it can be considered that the non-linear error caused by the ellipticity of the two beams is nearly superimposed. R is a statistical measure that quantifies the extent to which a regression equation accurately represents the variability in the data being analyzed. A higher R value indicates a better fit between the regression model and the data, indicating that the model can explain more of the variation in the dependent variable.

Through this simulation, it can be found that among the various error factors in heterodyne interferometry based on one single-mode PMF, the temperature, T, has no effect on the maximum value of the nonlinear error, and the phase error introduced by the temperature change in the PMF can be eliminated by placing the reference signal after the PMF. The nonlinear error caused by the ellipticity $\rho_{1}$, $\rho_{2}$ and the non-orthogonal degree β of the dual-frequency laser is much smaller than the nonlinear error caused by the angular misalignment error θ of the PMF. When the angular misalignment error is 5°, the nonlinear error is as high as 2.9469 nm. Therefore, the angular misalignment error θ of the PMF is the main factor causing its nonlinear error. By optimizing the accuracy of the alignment scheme of the PMF, the error of heterodyne interferometry based on one single-mode PMF can be significantly reduced, and the measurement accuracy can be improved. According to the simulation results, when the angular misalignment error of the PMF is 2.87°, the nonlinear error is 0.9974 nm, and when it is 0.25°, the nonlinear error is 9.60 pm. For the first time, we provided a goal for the optimization of the alignment scheme and the accuracy of the PMF to a sub-nanometer level or even better, through simulation. In actual measurement, the angular misalignment error of the PMF needs to be smaller than 2.87° to achieve sub-nanometer interference accuracy, and smaller than 0.25° to make the influence smaller than ten picometers.

## 4. Conclusions

In the heterodyne interferometry system based on one single-mode PMF, the ellipticity $\rho_{1}$, $\rho_{2}$ and non-orthogonality β of the dual-frequency laser, the angular misalignment error θ of the PMF, and the temperature, T, will all cause nonlinear errors. In this paper, a heterodyne interferometry error model based on one single-mode PMF is innovatively constructed, and the above five influencing factors are analyzed separately. The simulation results show that the angular misalignment error θ of the PMF has the greatest impact on the nonlinear error in the measurement. When the angular misalignment error reaches 5°, the nonlinear error is as high as 2.9469 nm. The temperature, T, has no effect on the maximum value of the nonlinear error, and the phase error introduced by the temperature change in the PMF can be eliminated by placing the reference signal after the PMF. Therefore, it is necessary to pay special attention to the coupling alignment of the PMF during actual system design. When the alignment accuracy of the PMF is improved, the measurement error will be significantly reduced, and the measurement accuracy can be greatly improved. For the heterodyne interferometry system based on one single-mode PMF, we provided a target for the optimization of the alignment scheme and the improvement of the precision of the PMF to the sub-nanometer level or even better, through simulation, for the first time. In actual measurement, the angular misalignment error of the PMF θ needs to be smaller than 2.87° to achieve sub-nanometer interferometric precision, and smaller than 0.25° to make the influence smaller than ten picometers. On the other hand, while the mathematical model proposed in this paper is applicable to the error analysis of heterodyne interferometry based on one single-mode PMF, it can also provide guidance for designing similar interferometric length measurement systems with dual-frequency laser, coupling optical elements. In terms of error compensation, this paper not only provides guidance for optical methods through simulation, and points out the importance of alignment of PMF and the basis for alignment requirements, but also provides formulas for numerical methods to compensate for errors and calculate non-linear errors produced by multiple factors.

## Figures and Tables

**Table 1:** Material parameters of PMF.

	Young’s Modulus/N·m^−2^	Poisson’s Ratio/1	Coefficient of Thermal Expansion/ K^−1^	Refractive Index/1
Core region	7.830 × 10^10^	0.186	1.06 × 10^−6^	1.456
Stress region	7.830 × 10^10^	0.186	2.15 × 10^−6^	1.444
Cladding	7.830 × 10^10^	0.186	0.54 × 10^−6^	1.444

**Table 2:** Nonlinear errors introduced by various influencing factors.

Angle/°	Nonlinear Error Introduced by $\rho_{1}$ **/nm**	Nonlinear Error Introduced by β/nm	Nonlinear Error Introduced by θ/nm
1	0.0153	0.0153	0.1225
2	0.0614	0.0612	0.4877
3	0.1383	0.1375	1.0884
4	0.2462	0.2438	1.9133
5	0.3854	0.3796	2.9469

## Data Availability

Data underlying the results presented in this paper are not publicly available at this time but maybe obtained from the authors upon reasonable request.
